# Supplementary material for: Quartet DNA reference materials and datasets for comprehensively evaluating germline variant calling performance
Source: Genome Biol. 2023 Nov 27;24:270. doi: 10.1186/s13059-023-03109-2 (PMC10680274; doi:10.1186/s13059-023-03109-2)
Supplement: Supplementary file 1 — Additional file 1. Supplementary figures. [file 13059_2023_3109_MOESM1_ESM.docx]

**Additional file 1: Supplementary figures**

**Quartet DNA reference materials and datasets for comprehensively evaluating germline variants calling performance**

Ren L et al.

*Genome Biology*


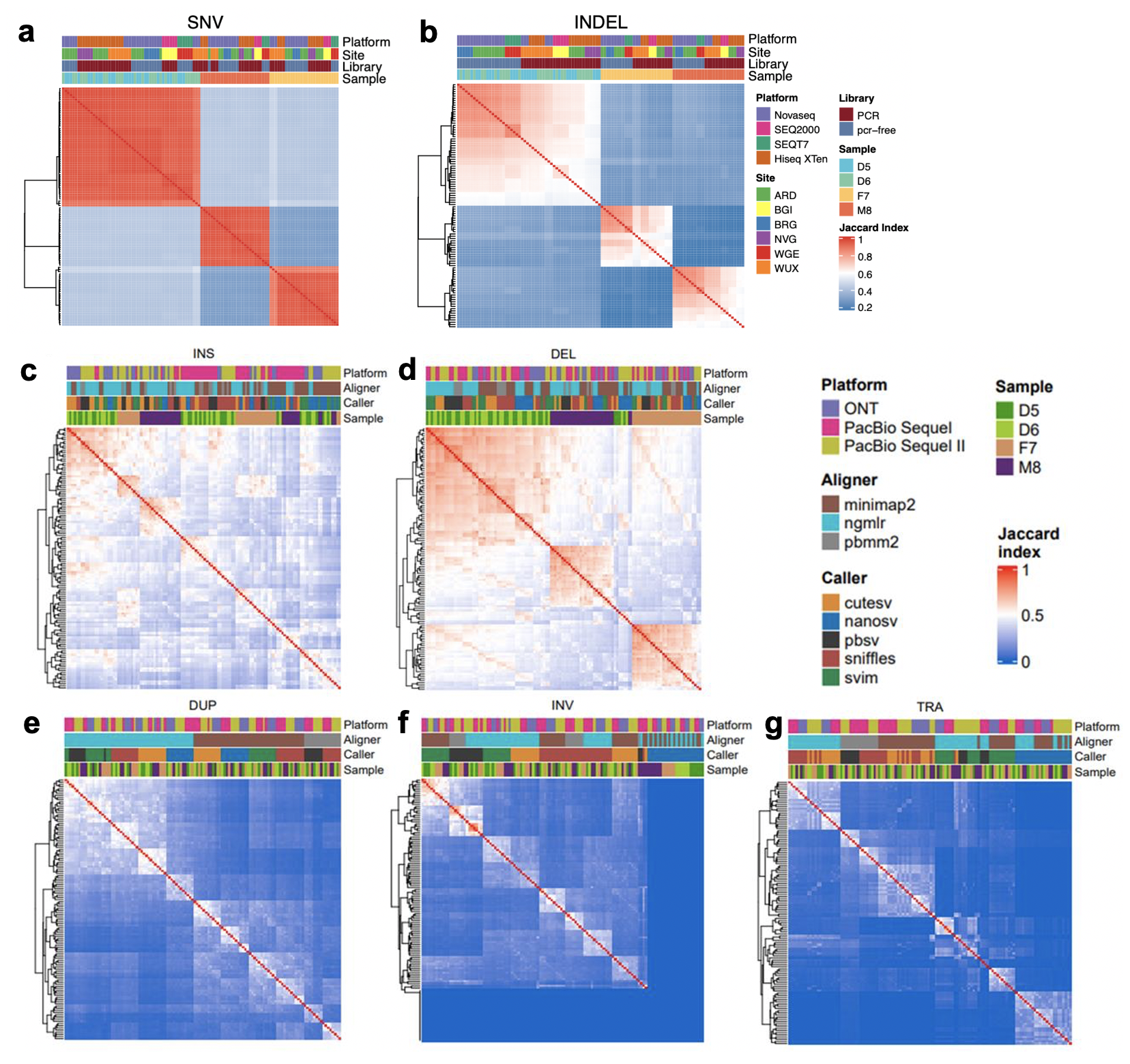


**Fig. S1.** Pairwise comparison of (**a**) SNVs, (**b**) small indels (**c**) insertions, (**d**) deletions, (**e**) duplications, (**f**) inversions, and (**g**) translocations called from short-read and long-read WGS call sets used to establish benchmark calls. Color of each cell corresponds to Jaccard index (the number of shared variants divided by the union of two call sets), with high similarity in red and low similarity in blue.


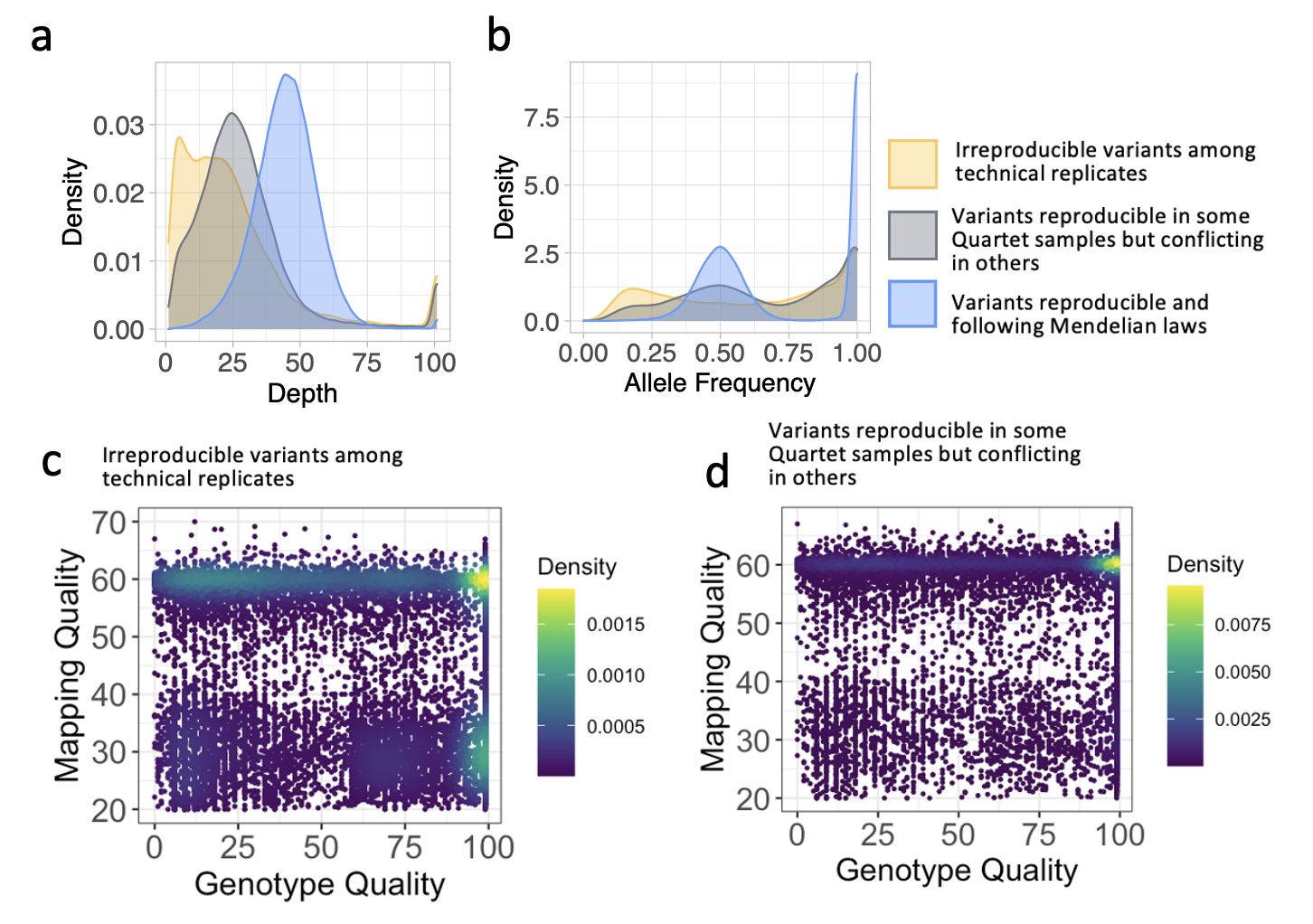


**Fig. S2.** Density plots show differences in (**a**) sequencing depth, (**b**) allele frequency, (**c** and **d**) genotype quality and mapping quality among irreproducible variants among technical replicates, variants reproducible in some Quartet samples but conflicting in others and variants reproducible and following Mendelian laws.


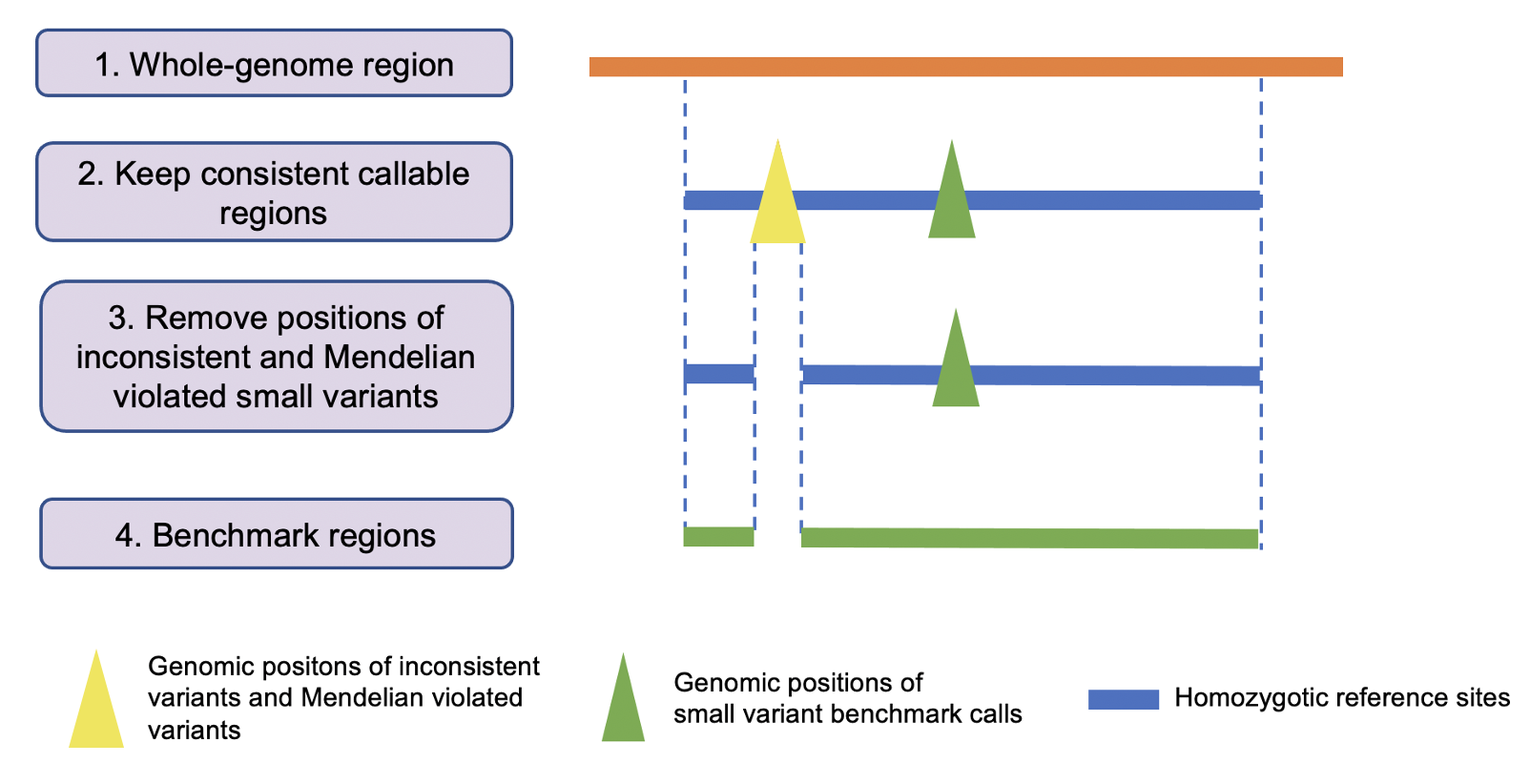


**Fig. S3.** Workflow of defining benchmark regions for small variants.

**
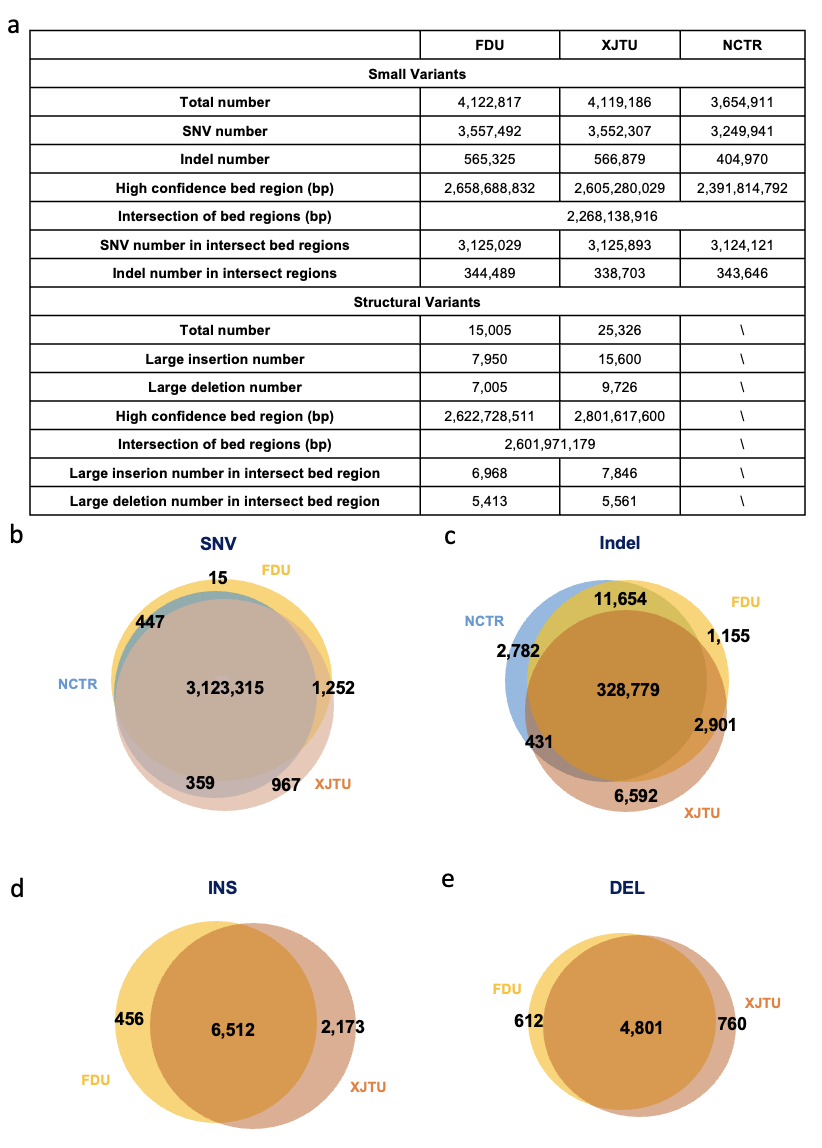
**

**Fig. S4.** Comparison of three benchmark sets from FDU (our study using short reads mapping for small variant benchmark calls and long reads mapping for structural variant benchmark calls across multiple technical replicates and incorporating Mendelian checking), NCTR (Pan et al. using short reads mapping across multiple bioinformatical tools^33^), and XTJU (Jia et al. using short reads mapping, long reads mapping and assemblies^34^). (**a**) Statistics of small variant benchmark sets, (**b**) Venn diagram of SNV benchmark sets, (**c**) Venn diagram of indel benchmark sets, (**d**) Venn diagram of DEL benchmark sets, and (**e**) Venn diagram of INS benchmark sets.


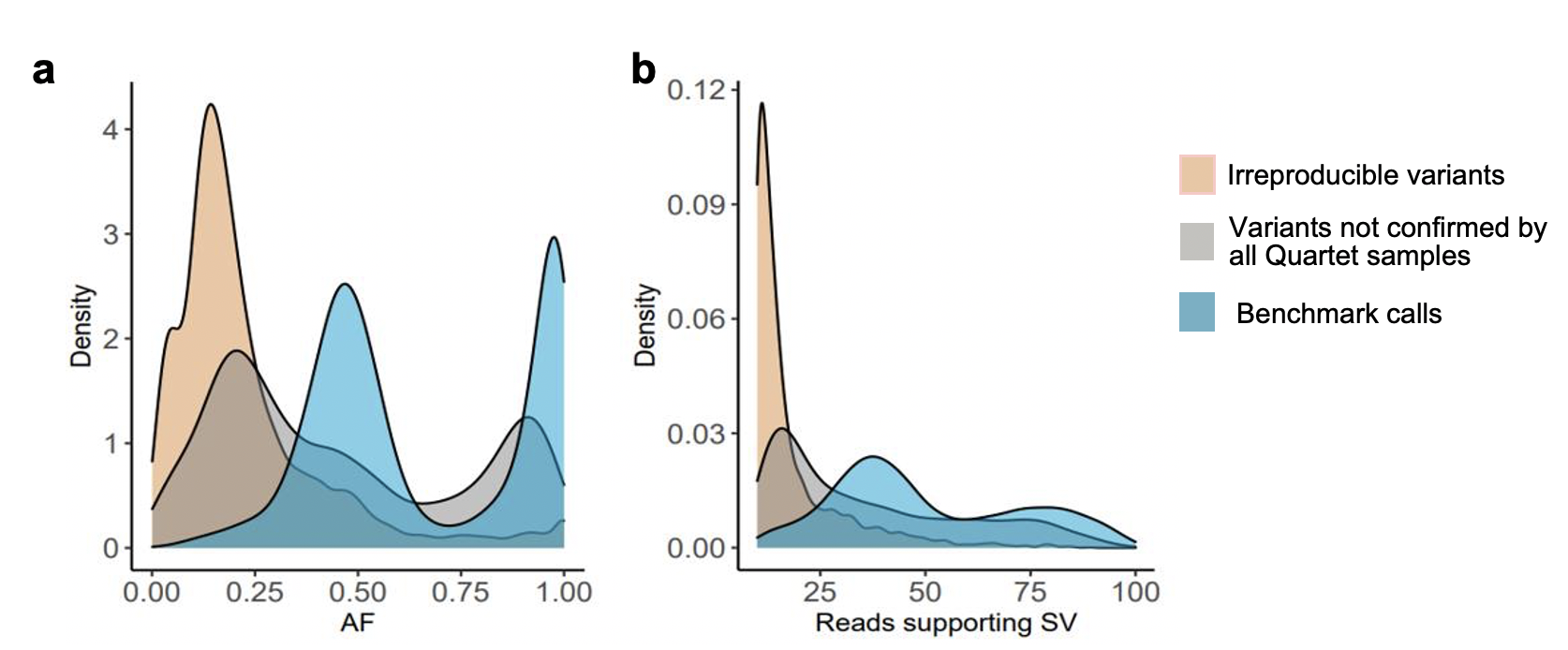


**Fig. S5.** Density plots show differences in (**a**) allele frequency and (**b**) reads supporting SV between irreproducible SVs, reproducible but Mendelian discordant SVs and reproducible and Mendelian concordant SVs.


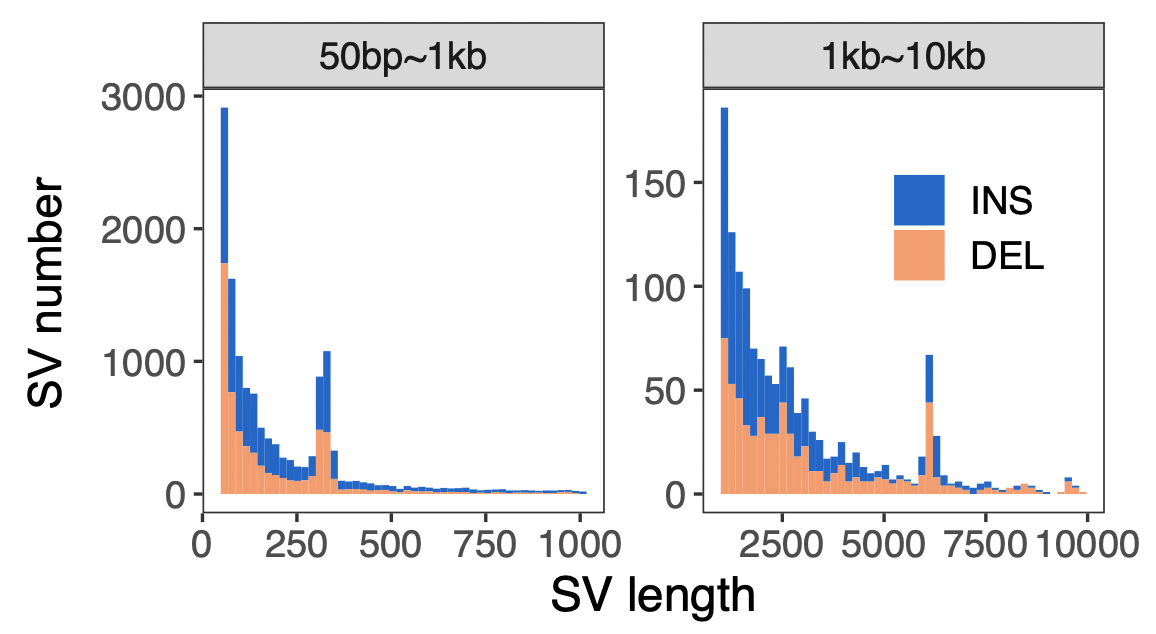


**Fig. S6.** Length distribution of SV benchmark calls for the Quartet DNA reference samples.


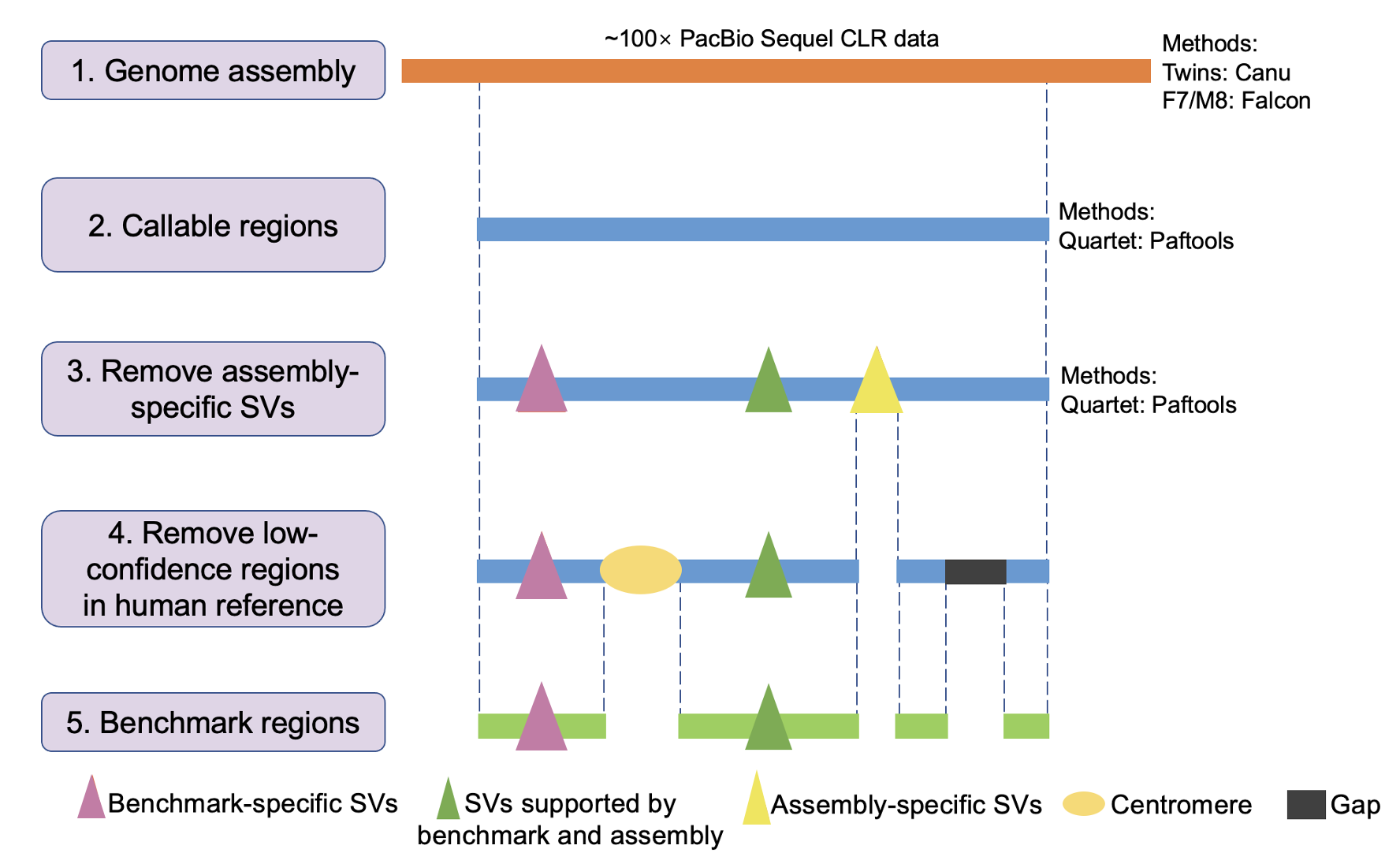


**Fig. S7.** Workflow of defining benchmark regions for structural variants.


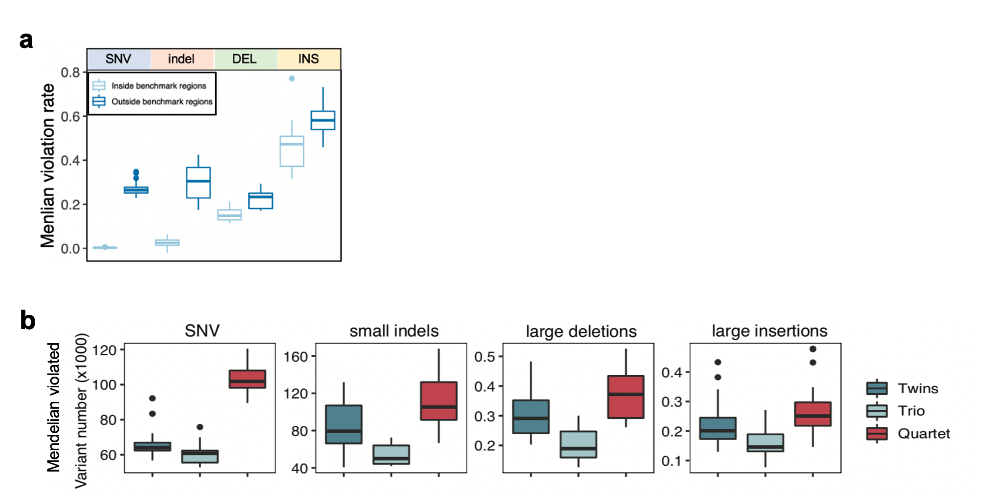


**Fig. S8.** **(a)** Comparison of Mendelian violation rate inside and outside the benchmark regions across different variant types. **(b)** Discordant variants detected by twins (D5 and D6), Mendelian discordant variants detected by trios (D5-F7-M8 and D6-F7-M8), and Mendelian discordant variants detected by Quartet family (D5-D6-F7-M8).


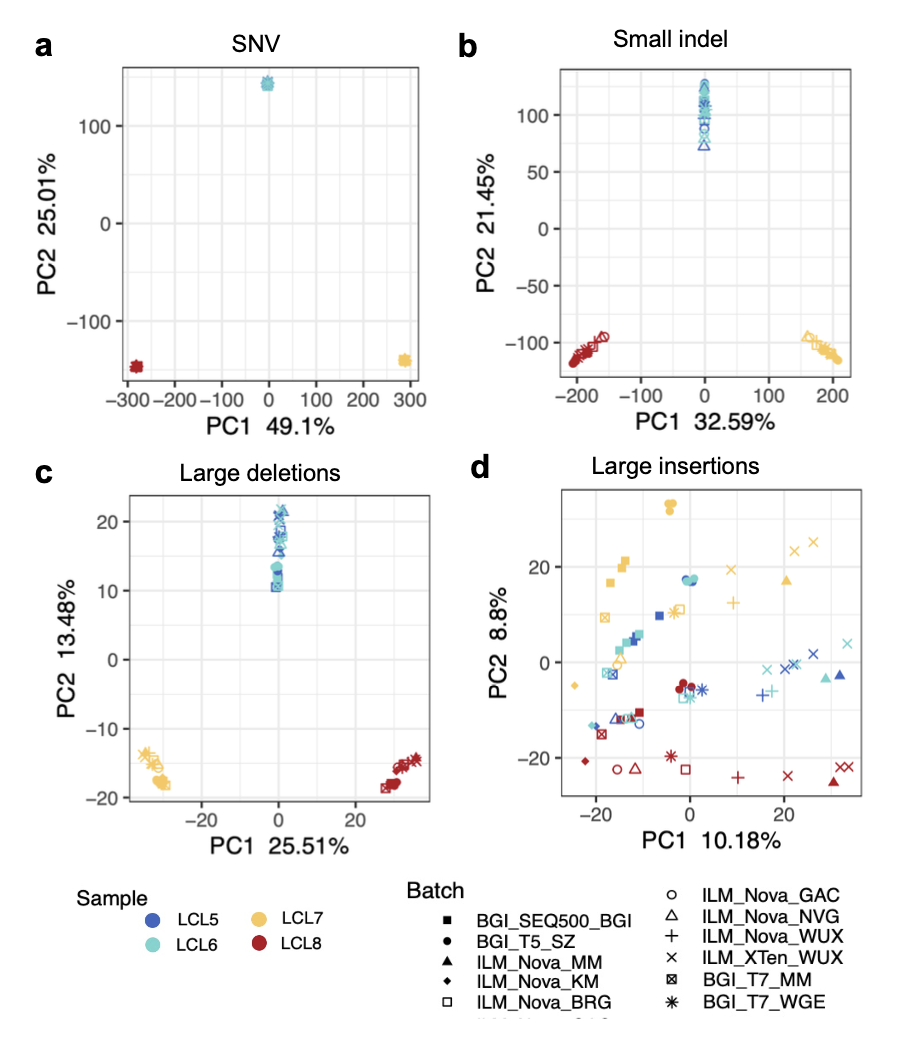


**Fig. S9.** The scatterplot of the first two eigenvectors generated from PCA displayed clustering of the Quartet samples. Four different variant types from 11 batches short-read sequencing datasets are shown as PCA plots. (**a**) SNVs; (**b**) Small indels; (**c**) Large deletions; and (**d**) Large insertions.


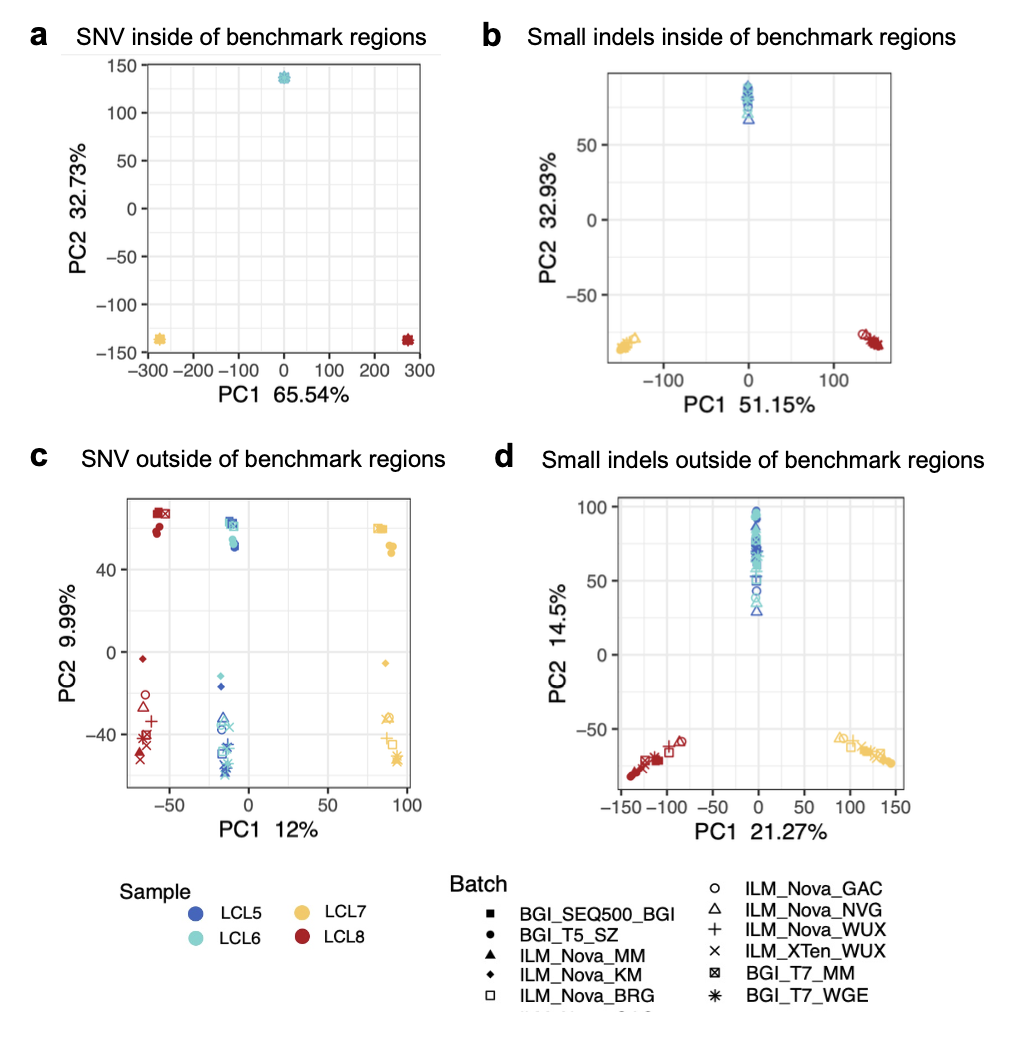


**Fig. S10.** Variants called outside the benchmark regions showed more severe batch effects than variants called inside the benchmark regions. Small variants called inside and outside of benchmark regions from 11 batches of short-read sequencing datasets are shown as PCA plots. (**a**) SNVs called inside the benchmark regions; (**b**) Small indels called inside the benchmark regions; (**c**) SNVs called outside the benchmark regions; and (**d**) Small indels called outside the benchmark regions.
