# Supplementary material for: Quartet DNA reference materials and datasets for comprehensively evaluating germline variant calling performance
Source: Genome Biol. 2023 Nov 27;24:270. doi: 10.1186/s13059-023-03109-2 (PMC10680274; doi:10.1186/s13059-023-03109-2)
Supplement: Supplementary file 7 — Additional file 7. Review history. [file 13059_2023_3109_MOESM7_ESM.docx]

Review History

**First round of review**

**Reviewer 1**

**Were you able to assess all statistics in the manuscript, including the appropriateness of statistical tests used?**

Yes, and I have assessed the statistics in my report.

**Comments to author:**

The paper of Ren et al describes a new reference material (RM) consisting of LCL DNA from a human quartet aimed to providing a resource for benchmarking DNA sequencing technologies, workflows, and bioinformatic methods, among other applications. This material is a parallel to the RMs developed by the Genome-in-a-Bottle (GiaB) consortium and US NIST. The distinctive feature of this material is the inclusion of homozygotic twin offspring which can provide enable more extensive Mendelian consistency checking when developing a truth set for variant calls. Additionally, they develop a truth set for the RM by an extensive combination of sequencing libraries from multiple platforms, short-read and long read, including also technical replicates, enabling the development of an ensemble approach that takes advantage of reproducibility of calls across the libraries. While GiaB/NIST RMs are excellent and in continuous improvement, the set of available RMs is till limited and additional materials with new characteristics would be welcome by the life science tools and clinical communities.

The manuscript is well written and very comprehensive in general. But a few items still need clarification or correction.

1. Page 2, line 41: "a DNA reference material" (not "the")
2. Across several places in the manuscript, new terms/concepts are introduced but is not clear where they are defined. Examples:
a. Page 6, Line 160 & Page 23, Line 578 : "voting" - how is this voting performed? This is explained in the methods, so please refer to the Methods section (cf. Methods).
b. Page 6, Line 171 & Page 7, line 201: "callable regions" - refer to Methods. (do this at least for the first instance of a term)
3. Page 6, line 167 - Could GATLK VQSLOD as a filtration method be effective? This was not used to construct the truth set, so I wonder how this compares to the SVM develop in this work.
4. Page 7, line 178. Not clear at all where these somatic variants come from. I understand that LCL may accumulate cell line somatic variants, which will not be present in data from blood samples, but those that are found both in blood and LCL DNAs, aren't they simply germline and not somatic? Please clarify.
5. Page 7, Line 185 - it is not clear what these two numbers refer to (98.7% vs 95.0%). Please clarify.
6. Page 7, Lines 193-195: Were the PMRA array calls not present in NGS within the callable regions? If so, I would think these array calls are artifacts rather than real.
7. Page 8, Lines 217-218. This fixed size window may be okay for large events, but smaller true separate calls, say of 50-200bp, would be merged unnecessarily. Would a fractional approach (e.g. 10% of the length of the call) or tiered approach (based ion size of calls) be better? I feel this can create FN/FPS when benchmarking SVs.
8. Page 10, lines 274-276: The Methods section does not include details on how these calls were made and what parameters were used. E.g.? Does the GATK HC used VQSLOD filtering. Does Sentieon calls used their ML filtering method? Do RTG call set used WGS AVR model and filtered at certain threshold? This is necessary.
9. Page 12, lines 355365; Page Page 44, Lines 968-976 - these RNAseq variant calls likely include significant FP artifacts. The GATK RNAseq variant calling protocol is not an accepted "best practices," as they indicate in their webpage. Others have found that removal of alignment and base-quality artifacts is needed to improve the quality of variant calls made from RNAseq data with tools like Opossum (cf. ), and a recent preprint demonstrates that even this includes unacceptable levels of FP calls (cf. https://doi.org/10.1101/2022.10.16.512451), and presents a new model for DeepVariant with significantly improved performance for this application. Therefore, any comments on the role of RNA editing in these results are unwarranted (it is known that RNA editing is relatively modest as a biological process). I suggest the authors if they want to make any points with RNAseq derived calls they develop a proper call set with more current methods.
10. Page 15, Lines 408-409 - what sample would be better suited? Probably one of the Twins given their calls benefit more from Mendelian inconsistency filtering than their parents.
11. Page 15, Line 416 - this is still expensive in the long run for clinical labs given reimbursement pressures, and I am, not sure is justified. An alternative approach would be the use of internal controls in every sample such as "sequins" (doi: 10.1038/s41467-019-09272-0) as this approach would allow performance and batch effect normalization as well and could be more cost effective. Can the authors compare their suggestion to this approach?
12. Page 28, Line 616: what metrics are referred to? Unclear. And if this is relevant to the paper, please include here or in methods.
13. Page 32: Table 2 is not mentioned in the main text! Either remove or add content that refers to these results.
14. Page 39, Line 838: what "most" mean; be specific.
15. Page 42, Line 909: These regions may still be variable but not reachable by short reads. How do we know?
16. Page 42, Line 913: How about consistency with SV calls. since some of these variants sites could be overlapped by an indel.
17. Page 45, Line 994: does hap.py uses the option to use "vcfeval" as the comparation engine (it should be used like this).

A few general comments: the consensus (voting) approach tends to create FNs. The "confident invariant regions" are derived from GATK using depth and read quality analysis, but as mentioned, some regions may not be tractable to short reads (e.g. PSM2 gene exons that are almost identical to its pseudogene). I would like to see an explicit approach to assess for FNs in the benchmark regions, perhaps using the long read platforms for small calls calling and analyzing any missing calls in the small variant truth set for candidate FNs. Further, have the authors considered to develop T2T assemblies for the quartet as an alternative approach to generate truth set that don't rely on aligners and variant callers? Given this is possible now form very similar source data it would be a remiss not to at least discuss this and their plans.

**Reviewer 2**

**Were you able to assess all statistics in the manuscript, including the appropriateness of statistical tests used?**

Yes, and I have assessed the statistics in my report.

**Comments to author:**

This study deals with the question of technical error in calling genomic variants due to different sequencing platforms. The authors use lymphoblastoid cell lines from a nuclear family of 4 members (father, mother, monozygotic twin 1, monozygotic twin 2.

The manuscript is full of interesting numbers, but it is written in a suboptimal way; the messages are not spelled out, and the conclusions are obscure.

1. The abstract is uninteresting; what is the message? why was the study done? which question did the authors tried to answer?

2. What is the definition of benchmarked regions ? what is outside of the benchmark regions?

3. This reviewer suggests to repeat the analyses using the T2T CHM13 reference sequence.

4. This reviewer suggests to attempt haplotype sequence for the entire chromosomes using the long-read sequences and the tools developed by the T2T investigators.

5. The discrepancy between the RNASeq and DNASeq is very interesting, and it should be presented more prominently.

6. The nature of the somatic variants should be analyzed and discussed.

7. The nature of the variants due to cell culture should be analyzed and discussed.

8. For people that use the exome as a diagnostic tool, it would interesting to know the data analysis within the coding regions and the splice junctions of the protein-coding genes.

**Authors Response**

**Point-by-point responses to the reviewers’ comments:**

**Reviewer #1:**

The paper of Ren et al describes a new reference material (RM) consisting of LCL DNA from a human quartet aimed to providing a resource for benchmarking DNA sequencing technologies, workflows, and bioinformatic methods, among other applications. This material is a parallel to the RMs developed by the Genome-in-a-Bottle (GiaB) consortium and US NIST. The distinctive feature of this material is the inclusion of homozygotic twin offspring which can provide enable more extensive Mendelian consistency checking when developing a truth set for variant calls. Additionally, they develop a truth set for the RM by an extensive combination of sequencing libraries from multiple platforms, short-read and long read, including also technical replicates, enabling the development of an ensemble approach that takes advantage of reproducibility of calls across the libraries. While GiaB/NIST RMs are excellent and in continuous improvement, the set of available RMs is till limited and additional materials with new characteristics would be welcome by the life science tools and clinical communities.

**Response:**

Thank you very much for your insightful comments. We greatly appreciate your recognition of the value of the Quartet DNA reference materials to the community.

The manuscript is well written and very comprehensive in general. But a few items still need clarification or correction. 
**Response:**

Thank you very much for your positive comments. We have addressed all your questions and concerns below.

1.	Page 2, line 41: "a DNA reference material" (not "the")

**Response:**

Thank you for pointing out this. We have corrected the grammatical error in the revised manuscript (Page 2, Line 43).

2.	Across several places in the manuscript, new terms/concepts are introduced but is not clear where they are defined. Examples:
a.	Page 6, Line 160 & Page 23, Line 578 : "voting" - how is this voting performed? This is explained in the methods, so please refer to the Methods section (cf. Methods).

**Response:**

We have revised “Integration of small variant benchmark variants” in the Methods section to make this clearer (please see Page 37, Line 876 to 917). We have referred to the Methods section in the main text, as suggested by the reviewer.

b.	Page 6, Line 171 & Page 7, line 201: "callable regions" - refer to Methods. (do this at least for the first instance of a term)

**Response:**

We have added explanation of callable regions to make this clearer (please see Page 8 Line 208 to 212).

3.	Page 6, line 167 - Could GATLK VQSLOD as a filtration method be effective? This was not used to construct the truth set, so I wonder how this compares to the SVM develop in this work.

**Response:**

When establishing the benchmark variants, we did not use any filtration method to filter variants. Because a true variant can have low quality, but it can still be consistently detected by technical replicates and follow Mendelian laws.

We have revised the figure to make it clearer in Page 47 Line 1120-1123. Before conducting Mendelian analysis, the variants in any Quartet sample that did not pass the voting process or contains no information was removed. For example, if the voting results of a variant site were [“0/1”, “0/1”, “Conflict”, “1/1”] for [D5, D6, F7 and M8], and F7 did not pass the voting process at this variant locus, the variant would be considered unreliable, even if D5, D6 and M8 had consistent voting results at this variant locus. Similarly, if the voting results of a variant site were [“0/1”, “0/1”, “./.”, “1/1”], indicating that F7 had no reads coverage and no information at this variant locus, this variant would also be considered unreliable and would be removed. In total, 412,054 sites with such conditions were removed. We compared the quality of three types of variants in Supplementary Figure 2: (1) irreproducible variants among technical replicates, (2) variants reproducible in some Quartet samples but conflicting in others, and (3) variants reproducible and following Mendelian laws.

To compare GATK Variant Quality Score Recalibration (VQSR) and the SVM developed in this study, we used the recommended procedures and settings (https://gatk.broadinstitute.org/hc/en-us/articles/4402736812443-Which-training-sets-arguments-should-I-use-for-running-VQSR-) to run VQSR for filtering variants in the Quartet samples. GATK VQSR exhibited higher precision and lower recall for SNVs, and both lower precision and recall for Indels (Figure R1). GATK VQSR filtered out more variants, resulting in an average of 3.3 million SNVs and 82 thousand Indels, whereas the SVM retained more variants, with 3.88 million SNVs and approximately 92 thousand Indels, which indicated that GATK VQSR is more stringent in filtering, leading to the exclusion of more true positives.

Figure R1. The precision, recall and F1-score of the variants were compared with benchmark calls both before and after filtration using SVM or GATK VQSR.

4.	Page 7, line 178. Not clear at all where these somatic variants come from. I understand that LCL may accumulate cell line somatic variants, which will not be present in data from blood samples, but those that are found both in blood and LCL DNAs, aren't they simply germline and not somatic? Please clarify.

**Response:**

Thank you for the comments and suggestions. The variants found in both blood and LCL DNAs, but Mendelian inconsistent within Quartet family, are somatic variants that have accumulated over a lifetime. In our analysis, we found a total of 7,329 Mendelian inconsistent variants and tried to explain where they come from. Among them, 4,761 were located in the benchmark regions. We manually checked their alignment using IGV, and found 3,221 were true. We then found 1,034 of those validated variants overlap with large deletions (>50 bp), which were misclassified by trio analysis software, which assumes that all variants are inherited in a diploid manner (which was explained in our response to comment #16).

We then compared the remaining 2,187 variants with the variants detected from the blood DNA samples to determine whether these variants were *de novo* germline mutations or arose during cell culture. These mutations are divided into four sources:

(1) Pre-twining germline *de novo* variants: These variants refer to genetic changes that occur in the germ cells before the embryo splits to form monozygotic twins. They are shared between monozygotic twin daughters. For example, the genotype may be “0/0” for the parents and “0/1” for the identical twins. We found 95 pre-twining germline *de novo* variants shared by Quartet-D5 and Quartet-D6. These variants can be detected in both LCL cell lines and blood samples of the twins.
(2) Postzygotic germline *de novo* variants: These variants refer to genetic changes that occur after the embryo splits, resulting in unique in each individual twin. For example, the genotype may be “0/0” for the parents, “0/0” for one twin, and “0/1” for the other twin. We found 1 postzygotic germline *de novo* variant that was unique to Quartet-D6. This variant can be detected from LCL cell lines and blood samples of Quartet-D6.
(3) Somatic variants accumulated during a lifetime: These variants are Mendelian inconsistent and can be detected in both LCL cell lines and blood samples. For example, the genotype may be “0/1” for Quartet-F7, “1/1” for Quartet-M8, and “0/0” for Quartet-D5 and Quartet D6. We found a total of 1,532 such variant loci across all four Quartet family members.
(4) Somatic variants accumulated from cell culture: These variants are uniquely detected in LCL cell lines but not blood samples. For example, the genotype may be “0/0” in blood samples, while “0/1” in LCL samples. We found a total of 559 variants in Quartet family.

5.	Page 7, Line 185 - it is not clear what these two numbers refer to (98.7% vs 95.0%). Please clarify.

**Response:**

These two numbers refer to the average validation ratio of SNVs (98.7%) and small indels (95.0%) in the benchmark sets of four Quartet samples validated by PacBio CCS. We have revised the sentence to make it clearer (Page 8 Line 191).

6.	Page 7, Lines 193-195: Were the PMRA array calls not present in NGS within the callable regions? If so, I would think these array calls are artifacts rather than real.

**Response:**

Yes, the PMRA SNP array calls that are not present in NGS are located within the callable regions. We used the PMRA probes within the benchmark regions to verify the benchmark variants integrated from NGS data. We manually examined the alignment of these distinct genotypes in NGS using IGV. Seven of them were clearly identified as misalignment artifacts of NGS, and therefore, we excluded them from the benchmark variants.

7.	 Page 8, Lines 217-218. This fixed size window may be okay for large events, but smaller true separate calls, say of 50-200bp, would be merged unnecessarily. Would a fractional approach (e.g. 10% of the length of the call) or tiered approach (based ion size of calls)  be better? I feel this can create FN/FPS when benchmarking SVs.

**Response:**

The reason for selecting a fixed threshold to compare or merge SVs from different SV callsets is as follows: SV merging can be approached from various perspectives, such as overlap ratio, sequence similarity, or distance between breakpoints. However, each method has its own advantages and disadvantages, as discussed in references: PMID:31729472 and PMID:36575487.

During the construction of our SV benchmark set, we evaluated the usage of SUPVIVOR (PMID: 28117401), a widely used tool for merging SVs based on the distance between breakpoints (default: 1kb). However, we encountered issues with SURVIVOR, such as the merging of different types of SVs into a single SV. We were unable to merge SVs based on sequence similarity, like NIST (PMID: 32541955), due to certain callers (e.g., NanoSV and SVIM) not providing variant sequences in their output.

After testing Jasmine, we found that it met our requirements with its user-friendly interface and detailed output. Jasmine employs an improved minimum spanning forest algorithm to merge SVs from different callers. SVs of the same type are merged when the distance between their breakpoints meets the threshold (default value: 1000, calculated using Euclidean distance: [(start1-start2)^2 + (length1-length2)^2]^1/2 ≤ 1000). Furthermore, the size distribution of SVs in our benchmark set aligns with NIST, meaning the number of SVs decreases as their size increases, and we avoided excessive merging of SVs in the 50-200 bp range (Supplementary Fig. 6). Our benchmark set was also validated using multiple sequencing techniques (Supplementary Table 9). When benchmarking SVs, the newer version of Jasmine (<https://github.com/mkirsche/Jasmine>) supports the comparison or merging of SVs based on the variant length, with a default threshold set to 50% of the variant length.

8.	Page 10, lines 274-276: The Methods section does not include details on how these calls were made and what parameters were used. E.g.? Does the GATK HC used VQSLOD filtering. Does Sentieon calls used their ML filtering method? Do RTG call set used WGS AVR model and filtered at certain threshold? This is necessary.

**Response:**

We used variants calling results from our companion paper, which compared reproducibility across samples, platforms, aligners and callers (<https://doi.org/10.1186/s13059-021-02569-8>). We used raw variants calling results from all pipelines, and didn’t filter any potential false positives. Specifically, the GATK HC didn’t use VQSLOD filtering. Sentieon calls didn’t use their ML filtering method. RTG call set didn’t used WGS AVR model to filter at certain threshold. The default settings in bioinformatic tools are often recommended by developers as they are designed to be suitable for most scenarios. However, it is important to note that the default setting may not always be the optimal choice for every specific analysis. The reason not to filter the raw variants in this study was primarily due to the challenge of determining an appropriate threshold. Our study did not specifically investigate the impact of variant filtration thresholds or methods on the accuracy of variant calling.

9.	Page 12, lines 355-365; Page Page 44, Lines 968-976 - these RNAseq variant calls likely include significant FP artifacts. The GATK RNAseq variant calling protocol is not an accepted "best practices," as they indicate in their webpage. Others have found that removal of alignment and base-quality artifacts is needed to improve the quality of variant calls made from RNAseq data with tools like Opossum (cf. ), and a recent preprint demonstrates that even this includes unacceptable levels of FP calls (cf. <https://doi.org/10.1101/2022.10.16.512451>), and presents a new model for DeepVariant with significantly improved performance for this application. Therefore, any comments on the role of RNA editing in these results are unwarranted (it is known that RNA editing is relatively modest as a biological process). I suggest the authors if they want to make any points with RNAseq derived calls they develop a proper call set with more current methods.

**Response:**

Thank you for your comments and suggestions. We double-checked the results. In the previous analysis, we didn’t restrict our analysis to exon regions or coding sequences (CDS), resulting in a significantly higher number of called variants, approximately 57,000. In the revised manuscript, we restricted our analysis to the intersected genomic regions of benchmark regions, CDS regions and regions with a minimum of 3x coverage, following the methodology by Cook et al. (<https://doi.org/10.1101/2022.10.16.512451>) mentioned by the reviewer. We also compared the variants called by GATK HaplotypeCaller and DeepVariant using RNAseq models (Figure R2). On average, GATK detected 16,305 SNVs and 1,338 Indels, while DeepVariant detected 13,181 SNVs and 334 Indels in these intersected regions. DeepVariant yielded higher precision and recall than GATK for both SNVs and Indels. About 8.3% RNA variants from DeepVariant and 25.9 % from GATK in RNA-seq could not be validated by DNA small variant benchmark calls. When comparing false positives against known RNA editing sites in REDIportal (http://srv00.recas.ba.infn.it/atlas/), we observed that DeepVariant disregarded RNA editing event (17 out of 1,100), as mentioned by Cook et al., whereas GATK detected more RNA editing events (562 out of 4,234). This indicates that RNA editing does not have a significant contribution to the high level of inconsistency observed between variants identified from DNA and RNA sequencing datasets. Instead, the discrepancy is primarily attributed to technical artifacts.

Figure R2 (Figure 6 B, C in the revised manuscript). Evaluating variant calling accuracy from RNA by benchmark variants constructed from DNA data. (A) Precision and recall of variants called from GATK HaplotypeCaller and DeepVariant. (B) Composition of variant types in RNAseq variant call sets.

10.	Page 15, Lines 408-409 - what sample would be better suited? Probably one of the Twins given their calls benefit more from Mendelian inconsistency filtering than their parents.

**Response:**

Any of the four Quartet samples can be used for the assessment. If the regions and variants of interest are included within the defined benchmark regions, the performance of variant calling can be evaluated based on reference datasets. Because the benchmark regions for these Quartet samples are the same, any of the four Quartet samples can be suitable for this purpose.

11.	Page 15, Line 416 - this is still expensive in the long run for clinical labs given reimbursement pressures, and I am, not sure is justified. An alternative approach would be the use of internal controls in every sample such as "sequins" (doi: 10.1038/s41467-019-09272-0) as this approach would allow performance and batch effect normalization as well and could be more cost effective. Can the authors compare their suggestion to this approach?

**Response:**

Thank you for your comments and suggestions. Quartet DNA reference materials are examples of biological reference materials, while sequins are synthetic spike-in controls. These two types of reference samples differ in their origin, purpose, and characteristics. Biological reference materials accurately reflect the size and complexity of the genome, and are readily commutable to study samples. Synthetic spike-ins are artificially generated DNA sequences that are designed to mimic specific genomic regions or variants, which cannot be aligned to human reference genome. They serve as targeted representations of specific variations, thereby may not fully encompass the complexity of natural genomes. Synthetic spike-in can effectively assess the performance of quantitative genomic features, such as variant allele frequency (VAF) and copy number. By combing these two reference materials, researchers can obtain a more comprehensive and understanding of the performance of their sequencing datasets.

12.	Page 28, Line 616: what metrics are referred to? Unclear. And if this is relevant to the paper, please include here or in methods.

**Response:**

Thank you for pointing this out. We have added the definition of reproducibility between two variant call sets in the Methods section (Page 43 Line 1051-1059).

13.	Page 32: Table 2 is not mentioned in the main text! Either remove or add content that refers to these results.

**Response:**

Thank you for pointing this out. We have added content that refers to these results in Page 15 Line 402-405.

14.	Page 39, Line 838: what "most" mean; be specific.

**Response:**

“Most” refers to a minimum of 14 out of 27 (over 50%) call sets, taking into account replicates, labs, and library preparation methods. We have revised “Integration of small variant benchmark variants” in the Methods section to make this clearer (please see Page 37 Line 876 to 917).

15.	Page 42, Line 909: These regions may still be variable but not reachable by short reads. How do we know?

**Response:**

In this study, we used the GATK CallableLoci tools to extract callable genomic regions from 108 BAM files. The specific parameters used for this analysis were “—maxDepth 300 –maxFractionOfReadsWithLowMAPQ 0.1 –maxLowMAPQ 1 –minBaseQuality 20 –minMappingQuality 20 –minMappingQuality 20 –minDepth 10 –minDepthForLowMAPQ 10”. Approximately 89% of the genome are callable regions with high mapping quality and reasonable sequencing depth, neither too high nor too low. Other genomic regions where variants could not be accurately detected included reference genome bases with N (Ref_N, ~5.3%), regions with no read coverage (No Coverage, ~0.76%), regions with sequencing depth below 10x (Low Coverage, ~0.81%), regions with sequencing depth exceeding 300x (Excessive Coverage, ~0.1%, and regions with poor mapping quality (~3.9%) (Figure R3).

To establish benchmark regions, we defined them based on consensus across the 108 replicates. Additionally, regions displaying misalignment in NGS data were removed after validating the benchmark variants using PMRA and PacBio CCS reads (Page 8 Line 188-206).

Figure R3. The distribution of various genomic regions in 108 Quartet BAM files.

16.	Page 42, Line 913: How about consistency with SV calls. since some of these variants sites could be overlapped by an indel.

**Response:**

We excluded large deletions and insertions (>50 bp) and their flanking regions of 50 bp from the benchmark regions for the following reasons:

(1) In the Quartet family, 44.3% of small variants that overlap with large deletions exhibit Mendelian inconsistency, compared to only 0.3% in non-repetitive diploid genomic regions. Only 11.7% of small variants overlapping with large deletions represent true variants that have been misclassified as Mendelian violations. Among the 4,761 reproducible variants within technical replicates that did not follow Mendelian inheritance laws in the benchmark regions, 1,034 of them were found within large deletions. This misclassification occurs because trio analysis software assumes that all variants are inherited in a diploid manner and does not consider a specific variant where the father’s genotype is “0/1” and the mother’s genotype is “0/0” with a large deletion. Theoretically, in diploid genomes, the child’s genotype should be “0/0” or “0/1”. However, the actual genotype of the child is “1/1”, because the child inherited the variant from the father and the large deletion from the mother (Figure R4).

Figure R4. A schematic representation of co-inheritance of point mutations and large deletions. The child inherits a variant from one parent and a large segment deletion from the other parent, with the genotype conforming to Mendelian inheritance patterns.

(2) The number of small variants increases as the distance from the breakpoint decreases, and the proportion of variants that do not follow to Mendelian inheritance rules also increases. The number of variants and the proportion of Mendelian inconsistent variants stabilize beyond a distance of 500bp from breakpoint, with the Mendelian inconsistent rate decreasing from 0.6 to 0.1 (Figure R5).

Figure R5. (A) The number of small variants detected in 27 technical replicates for each of the four Quartet samples before and after insertion breakpoints, within a 50bp interval. (B) The proportion of Mendelian violated small variants before and after insertion breakpoints, within a 50bp interval. (C) The number of small variants detected in 27 technical replicates for each of the four Quartet samples before and after large deletion breakpoints, within a 50bp interval. (D) The proportion of Mendelian violated small variants before and after deletion breakpoints, within a 50bp interval.

17.	Page 45, Line 994: does hap.py uses the option to use "vcfeval" as the comparation engine (it should be used like this).

**Response:**

Thank you very much for your suggestion. Yes, hap.py uses the option to use “vcfeval” as the comparation engine. We have revised the sentences to make it clear (Page 44 Line 1069).

A few general comments: the consensus (voting) approach tends to create FNs. The "confident invariant regions" are derived from GATK using depth and read quality analysis, but as mentioned, some regions may not be tractable to short reads (e.g. PSM2 gene exons that are almost identical to its pseudogene). I would like to see an explicit approach to assess for FNs in the benchmark regions, perhaps using the long read platforms for small calls calling and analyzing any missing calls in the small variant truth set for candidate FNs. Further, have the authors considered to develop T2T assemblies for the quartet as an alternative approach to generate truth set that don't rely on aligners and variant callers? Given this is possible now form very similar source data it would be a remiss not to at least discuss this and their plans.

**Response:**

Thank you for your comments and suggestions. During the validation process of benchmark variants using PacBio CCS reads in Page 8 Line 188-194, we discovered variants specifically detected from PacBio CSS reads within the benchmark regions defined by NGS. Specifically, we identified 32,345 variants for Quartet D5 and D5, 33,894 variants for Quartet F7, and 36,637 variants for Quartet M8. When comparing these variants with those detected by both NGS and PacBio CCS reads, we observed that variants detected exclusively by PacBio CCS had lower genotype quality (Figure R6). Since we sequenced only one replicate of each Quartet sample using PacBio CCS, the variants detected solely by PacBio CCS will not be included in the benchmark variants without confirmation of additional evidence.

Figure R6. The density plot of genotype quality of variants detected only by PacBio CCS and variants detected by both PacBio CCS and NGS.

We have added a paragraph in the discussion about T2T assemblies for the Quartet DNA reference materials and benchmark variants consistency integrated from short reads and long reads (Page 16 Line 422-442 in the revised manuscript), as follows:

“As part of the Quartet Project, the main objective of our study is to provide the scientific community with genomic DNA reference materials that can be used to assess and improve the accuracy of germline variant calling. However, we acknowledge that the initial version of the small variant and structural benchmark sets for Quartet DNA reference materials does not include complex variants and genomic regions. This limitation arises from the challenges associated with mapping short reads to repetitive genomic regions and the potential mapping errors that can occur when calling structural variants solely through mapping approaches. In our companion study (Jia et al., <https://doi.org/10.1101/2022.09.08.504083>), we addressed these limitations by generating haplotype-resolved whole-genome assemblies for the monozygotic twin daughters. Decoding the complete genome of a diploid sample, compared to the complete hydatidiform mole (CHM13), presents more challenges. Nevertheless, we achieved high quality in the assemblies, with 76% of the chromosomal arms being gap-free from telomere to centromere. The updated benchmark regions increased to cover 92.43% of the GRCh38, including more complex variants and regions, through the integration of short reads, long reads, and haplotype assemblies. In this study, we compare benchmark sets integrated from three different sources: (1) multiple technical replicates generated from various platforms, laboratories, and batches analyzed using GATK best practices (as described in our study); (2) three batches of datasets called from multiple pipelines (as reported by Pan et al., [10.1186/s13059-021-02569-8](https://www.ncbi.nlm.nih.gov/pubmed/34980216)); and (3) haplotype-assemblies (as reported by Jia et al.) (**Supplementary Figure 4**). Our findings revealed that while benchmark variants integrated from variants called from short reads and long reads by mapping approaches may miss complex variants, there is high consistency in the benchmark variants within the overlapping benchmark regions, with 99.9% agreement for SNVs, 99.2% for indels, 93.5% for large deletions, and 88.7% for large insertions.”

**Reviewer #2:**

This study deals with the question of technical error in calling genomic variants due to different sequencing platforms. The authors use lymphoblastoid cell lines from a nuclear family of 4 members (father, mother, monozygotic twin 1, monozygotic twin 2.
The manuscript is full of interesting numbers, but it is written in a suboptimal way; the messages are not spelled out, and the conclusions are obscure.

**Response:**

We greatly appreciate the reviewer’s constructive comments and suggestions that helped us greatly improve our manuscript. Instead of focusing on technical errors in calling germline variants due to different sequencing platforms, our study focuses on developing genomic DNA reference materials and their corresponding reference datasets (benchmark variants), providing a standardized benchmark for evaluating the accuracy and comparability of variant calling results across different platforms, laboratories, batches, and analytical methods.

In our study, we first established four genomic DNA reference materials from immortalized cell lines derived from a family Quartet consisting of parent and monozygotic twins. The distinctive feature of this material suite is the inclusion of homozygotic twin offspring which can provide enable more extensive Mendelian consistency checking when developing a truth set for variant calls. If DNA reference materials are likened to “the exam”, the reference datasets can be considered “the right answers”, which can be used to identify true positive and false positive variants in a given test. We then developed small variant and structural variant benchmark variants by integrating multiple sequencing platforms, batches, and analytical methods. This comprehensive approach ensures a robust evaluation of variant calling accuracy. Lastly, we demonstrated three application scenarios for the Quartet DNA reference materials, including evaluating variant calling performance, identifying and mitigating batch effects in genomic sequencing, and evaluating variants called from mRNA and protein reference materials. Overall, our study provides a valuable resource that can enhance the accuracy, reliability, and comparability of variant calling in genomic research.

1. The abstract is uninteresting; what is the message? why was the study done? which question did the authors tried to answer?

**Response:**

Thank you for pointing out that the background in the abstract is not well-written in terms of describing the rationale for conducting our study.

DNA sequencing has been widely used in scientific research, as well as in the diagnosis and treatment selection for human diseases. However, the lack of reproducibility and consistency in variant call results obtained from different sequencing technologies, labs, or bioinformatic pipelines has hindered the clinical utility of sequencing applications. Genomic reference materials play a crucial role in ensuring the quality of genomic sequencing datasets, which have been extensively used for diagnostic performance assessment, proficiency testing, and assay development and optimization. Many genomic reference materials have been developed for this purpose, for example, seven germline reference materials released by the Genome in a Bottle (GIAB) consortium, led by the U.S. National Institute of Standards and Technology (NIST). However, previous studies have only used a single reference material to assess the performance of variant calling results, and these assessments were limited to genomic regions defined by benchmark regions (explained in our response to comment #2). This approach fails to consider the full extent of sequences generated and analyzed for a test genome. Consequently, relying solely on reference datasets to evaluate the accuracy of variant calling results is insufficient and incomplete, as it overlooks variants outside of the benchmark regions.

In cases where reference datasets are unavailable or do not contain the features of interest, alternative methods can be employed to assess performance. In our study, we developed a group of multi-sample reference materials derived from a Quartet family, consisting of parents and monozygotic twins. We proposed an approach to assess the performance of variant calling across the whole-genome region using the Mendelian inheritance laws observed in the monozygotic twins and their parents. While benchmark sets may not cover the entire whole genome region, it is still crucial to define them. This is because the assessment of precision can be done through the application of Mendelian inheritance laws (the proportion of detected variants are true), whereas benchmark variants are essential for evaluating the recall rate of a given test (the proportion of true variants should be called). The Quartet DNA reference materials have other unique features as well: (1) They enhance the representation of human genetic diversity in exiting genomic reference materials. This is because the family used in our study was recruited from the Fudan Taizhou cohort in Central China, encompassing genetic features from both Northern and Southern Chinese populations; (2) In addition to DNA reference materials, the Quartet Project also released RNA, protein, and metabolite reference materials derived from the same cell lines. This multiomics approach aligns with the central dogma of multiomics reference materials, enabling cross-omics validation of variant calls from multiomics datasets.

Based on the rationale described above, we have made revisions to the abstract (Page 2 Lines 38-42 in the revised manuscript) to clearly articulate the background of our research. The background of the revised abstract is as follows:

“Genomic DNA reference materials are widely recognized as essential for ensuring data quality in omics research. However, relying solely on reference datasets to evaluate the accuracy of variant calling results is incomplete, as they are limited to benchmark regions. Therefore, it is important to develop DNA reference materials that enable the assessment of variant detection performance across the entire genome.”

2. What is the definition of benchmarked regions? what is outside of the benchmark regions?

**Response:**

The Figure R7 illustrates the definition of benchmark regions. Benchmark regions represent a subset of the whole-genome region that undergo extensive characterization and validation, proving confidence in the genotypes called from these regions, regardless of whether they are variants or match the reference genome. However, complex or repetitive regions can present difficulties in accurately mapping reads, leading to an increased occurrence of false positive variants. As a result, these challenging regions are excluded from the benchmark regions, and variants in those regions are variants outside of the benchmark regions.

Figure R7. Schematic diagram for illustrating benchmark variants and regions.

When evaluating the performance of variant calling results using benchmark regions and variants, only variants within the benchmark regions are considered (Figure R8). Variants uniquely detected by the query set within benchmark regions are false positives (FP). Overlapping variants of the query set and benchmark calls are true positives (TP). Benchmark calls not detected by the query set are false negatives (FN). Precision describes the fraction of benchmark calls discovered in the query set. Recall describes the fraction of benchmark calls that have been detected. F1 score is the weighted average of precision and recall.

Figure R8. Schematic diagram for evaluating whole-genome variants calling performance.

We apologize for not initially providing an explanation of the meaning of benchmark regions for its first instance. We have added explanation of benchmark regions in the main text (Page 3 Lines 67 -70 in the revised manuscript), as follows:

“Benchmark regions are partial of whole-genome region that are well-characterized and validated. When evaluating the performance of variant calling results using reference materials based on a standalone reference material, only variants within these benchmark regions can be assessed.”

3. This reviewer suggests to repeat the analyses using the T2T CHM13 reference sequence.

**Response:**

The aim of our study is to provide the scientific community with genomic DNA reference materials and demonstrate their ability to assess and improve the performance of germline variant calling results. In our manuscript, we utilized the human reference genome GRCh38 to define their corresponding reference datasets, including benchmark variants and benchmark regions for small variants and structural variants. Recent advancements in sequencing technologies and analytical methods have led to the development of more complete and comprehensive human reference genomics, such as T2T CHM13 and the human pangenome (Wen-Wei Liao et al., Nature, 2023). However, GRCh38 remains the most widely used human reference genome within the sequencing community and offered with comprehensive annotation. Although this manuscript presents the initial version of the reference datasets for Quartet DNA reference materials, we will continuously update them to make them more comprehensive and suitable for a wider range of application scenarios. These updated reference datasets will be made available for download at the Quartet Data Portal website (https://chinese-quartet.org/#/reference-datasets/download).

We have added a paragraph in the discussion about T2T assemblies for the Quartet DNA reference materials and benchmark variants consistency integrated from short reads and long reads (Page 16 Line 422-442 in the revised manuscript), as follows:

“As part of the Quartet Project, the main objective of our study is to provide the scientific community with genomic DNA reference materials that can be used to assess and improve the accuracy of germline variant calling. However, we acknowledge that the initial version of the small variant and structural benchmark sets for Quartet DNA reference materials does not include complex variants and genomic regions. This limitation arises from the challenges associated with mapping short reads to repetitive genomic regions and the potential mapping errors that can occur when calling structural variants solely through mapping approaches. In our companion study (Jia et al., <https://doi.org/10.1101/2022.09.08.504083>), we addressed these limitations by generating haplotype-resolved whole-genome assemblies for the monozygotic twin daughters. Decoding the complete genome of a diploid sample, compared to the complete hydatidiform mole (CHM13), presents more challenges. Nevertheless, we achieved high quality in the assemblies, with 76% of the chromosomal arms being gap-free from telomere to centromere. The updated benchmark regions increased to cover 92.43% of the GRCh38, including more complex variants and regions, through the integration of short reads, long reads, and haplotype assemblies. In this study, we compare benchmark sets integrated from three different sources: (1) multiple technical datasets generated from various platforms, laboratories, and batches analyzed using GATK best practices (as described in our study); (2) three batches of datasets called from multiple pipelines (as reported by Pan et al., [10.1186/s13059-021-02569-8](https://www.ncbi.nlm.nih.gov/pubmed/34980216)); and (3) haplotype-assemblies (as reported by Jia et al.) (**Supplementary Figure 4**). Our findings revealed that while benchmark variants integrated from variants called from short reads and long reads by mapping approaches may miss complex variants, there is high consistency in the benchmark variants within the overlapping benchmark regions, with 99.9% agreement for SNVs, 99.2% for indels, 93.5% for large deletions, and 88.7% for large insertions.”

4. This reviewer suggests to attempt haplotype sequence for the entire chromosomes using the long-read sequences and the tools developed by the T2T investigators.

**Response:**

As part of the Quartet Project, which aims to ensure quality control of multiomics profiling encompassing DNA, RNA, protein, and metabolite analysis, this manuscript introduces Quartet DNA reference materials specifically focusing on the accuracy, reproducibility, and comparability of variant calling from short reads. In conjunction with this manuscript, we have conducted a companion study (Jia et al., https://doi.org/10.1101/2022.09.08.504083) to establish a more comprehensive set of reference datasets. In that companion study, we phased the long reads of the monozygotic twin daughters into paternal and maternal haplotypes and utilized long-read sequencing technology to generate highly complete and continuous haplotype-resolved whole-genome assemblies. By employing these haplotype-resolved assemblies, we were able to characterize variants that were underrepresented in the initial reference datasets from this manuscript, particularly those located in long repeat regions and complex structural variants.

5. The discrepancy between the RNASeq and DNASeq is very interesting, and it should be presented more prominently.

**Response:**

Thank you for your comments and suggestions. We double-checked the results. In the previous analysis, we didn’t restrict our analysis to exon regions or coding sequences (CDS), resulting in a significantly higher number of called variants, approximately 57,000. In the revised manuscript, we restricted out analysis to the intersected genomic regions of benchmark regions, CDS regions and regions with a minimum of 3x coverage, following the methodology by Cook et al. (<https://doi.org/10.1101/2022.10.16.512451>). We also compared the variants called by GATK HaplotypeCaller and DeepVariant using RNAseq models (Figure R9). On average, GATK detected 16,305 SNVs and 1,338 Indels, while DeepVariant detected 13,181 SNVs and 334 Indels in these intersected regions. DeepVariant yielded higher precision and recall than GATK for both SNVs and Indels. About 8.3% RNA variants from DeepVariant and 25.9 % from GATK in RNA-seq could not be validated by DNA small variant benchmark calls. When comparing false positives against known RNA editing sites in REDIportal (http://srv00.recas.ba.infn.it/atlas/), we observed that DeepVariant disregarded RNA editing event (17 out of 1,100), as mentioned by Cook et al., whereas GATK detected more RNA editing events (562 out of 4,234). This indicates that RNA editing does not have a significant contribution to the high level of inconsistency observed between variants identified from DNA and RNA sequencing datasets. Instead, the discrepancy is primarily attributed to technical artifacts.

Figure R9 (Figure 6 B,C in the revised manuscript). Evaluating variant calling accuracy from RNA by benchmark variants constructed from DNA data. (A) Precision and recall of variants called from GATK HaplotypeCaller and DeepVariant. (B) Composition of variant types in RNAseq variant call sets.

6. The nature of the somatic variants should be analyzed and discussed.
7. The nature of the variants due to cell culture should be analyzed and discussed.

**Response:**

Thank you for the comments and suggestions. We have analyzed *de novo* mutations of identical twins and somatic variants accumulated during lifetime and cell culture by comparing variants called from LCL and Blood DNA (Page 7 Line 179-187). In our analysis, we found a total of 7,329 Mendelian inconsistent variants and tried to explain where they come from. Among them, 4,761 were located in the benchmark regions. We manually checked their alignment using IGV, and found 3,221 were true. We then found 1,034 of those validated variants overlap with large deletions (>50 bp), which were misclassified by trio analysis software, which assumes that all variants are inherited in a diploid manner (which was explained in our response to comment #16).

We then compared the remaining 2,187 variants with the variants detected from the blood DNA samples to determine whether these variants were *de novo* germline mutations or arose during cell culture. These mutations are divided into four sources:

(1) Pre-twining germline *de novo* variants: These variants refer to genetic changes that occur in the germ cells before the embryo splits to form monozygotic twins. They are shared between monozygotic twin daughters. For example, the genotype may be “0/0” for the parents and “0/1” for the identical twins. We found 95 pre-twining germline *de novo* variants shared by Quartet-D5 and Quartet-D6. These variants can be detected in both LCL cell lines and blood samples of the twins.
(2) Postzygotic germline *de novo* variants: These variants refer to genetic changes that occur after the embryo splits, resulting in unique in each individual twin. For example, the genotype may be “0/0” for the parents, “0/0” for one twin, and “0/1” for the other twin. We found 1 postzygotic germline *de novo* variant that was unique to Quartet-D6. This variant can be detected from LCL cell lines and blood samples of Quartet-D6.
(3) Somatic variants accumulated during a lifetime: These variants are Mendelian inconsistent and can be detected in both LCL cell lines and blood samples. For example, the genotype may be “0/1” for Quartet-F7, “1/1” for Quartet-M8, and “0/0” for Quartet-D5 and Quartet D6. We found a total of 1,532 such variant loci across all four Quartet samples.
(4) Somatic variants accumulated from cell culture: These variants are uniquely detected in LCL cell lines but not blood samples. For example, the genotype may be “0/0” in blood samples, while “0/1” in LCL samples. We found a total of 559 variants in Quartet family.

8. For people that use the exome as a diagnostic tool, it would interesting to know the data analysis within the coding regions and the splice junctions of the protein-coding genes.
**Response:**

Thank you for your comments and suggestions. We have developed a quality control pipeline for whole-exome sequencing at the Quartet Data Portal (<https://chinese-quartet.org/#/seq-flow/app-store>, WES QC for Quartet). This application is designed to process Fastq files and VCF files as input, and provide detailed reports on the quality of raw reads, as well as the accuracy of variant calls. The accuracy of variants in the intersection of benchmark regions and exome capture regions can be evaluated by comparison with benchmark variants, and the variants outside of benchmark regions can be assessed by Mendelian checking among Quartet family.

To further support the research community, we have also made available benchmark variants specifically in CDS regions and exon regions. These benchmark variants can be accessed and downloaded from the Quartet Data Portal (<https://chinese-quartet.org/#/reference-datasets/download>). The coverage statistics for benchmark regions, including gene, exon and CDS regions are presented in Table 2.

As part of the Quartet project, this study focuses specifically on germline variant quality control. In addition to providing DNA reference materials, the Quartet Project has also released corresponding RNA, protein, and metabolite reference materials (Zheng Y et al., *Nature Biotechnology*, in press). One of the key “truths” is the central dogma of multiomics reference materials, which enables cross-omics validation. We have been working on developing reference datasets by validating them with two or more types of omics data, such as reference datasets of epigenetics and alternative splicing.

**Second round of review**

**Reviewer 1**

I assessed my own queries and also the queries of reviewer #2 as well. I conclude that the authors addressed satisfactorily all concerns/questions. The authors have satisfactorily addressed my questions, and the revised manuscript shows improvement. The analysis is thorough and detailed. This resource could be immensely valuable not only to the clinical laboratory community worldwide but also to developers of sequencing/genotyping technology, especially since the number of reference materials is very limited. While I cannot assess whether these materials will indeed be widely available based on the scientific content of this manuscript (as export laws and bilateral relations between countries may pose challenges), I hope the authors will make every effort to ensure global availability. If not, the impact of their publication could be diminished.

**Authors Response**

**Point-by-point responses to the reviewers’ comments:**

Reviewer #1: I assessed my own queries and also the queries of reviewer #2 as well. I conclude that the authors addressed satisfactorily all concerns/questions. The authors have satisfactorily addressed my questions, and the revised manuscript shows improvement. The analysis is thorough and detailed. This resource could be immensely valuable not only to the clinical laboratory community worldwide but also to developers of sequencing/genotyping technology, especially since the number of reference materials is very limited. While I cannot assess whether these materials will indeed be widely available based on the scientific content of this manuscript (as export laws and bilateral relations between countries may pose challenges), I hope the authors will make every effort to ensure global availability. If not, the impact of their publication could be diminished.

Response:
Thank you very much for your positive evaluation. We are very delighted that the reviewer found our response satisfactory. We have obtained permission from the Human Genetic Resources Administration of China (HGRAC) to make our reference materials and datasets publicly accessible without restrictions. The Quartet DNA reference materials have been certified by China’s State Administration for Market Regulation as the First Class of National Reference Materials and are extensively being utilized for proficiency testing and method validation. These materials have been utilized by over 100 institutions globally, including prestigious establishments such as the University of Helsinki in Finland, the European Infrastructure for Translational Medicine Research (EATRIS), among others. Accessing these materials is convenient through the Quartet Data Portal (<http://chinese-quartet.org>).
